# Supplementary material for: Proteome-Wide Identification of RNA-dependent proteins and an emerging role for RNAs in Plasmodium falciparum protein complexes
Source: Nat Commun. 2024 Feb 14;15:1365. doi: 10.1038/s41467-024-45519-1 (PMC10866993; doi:10.1038/s41467-024-45519-1)
Supplement: Supplementary file 1 — Supplementary Information [file 41467_2024_45519_MOESM1_ESM.pdf]

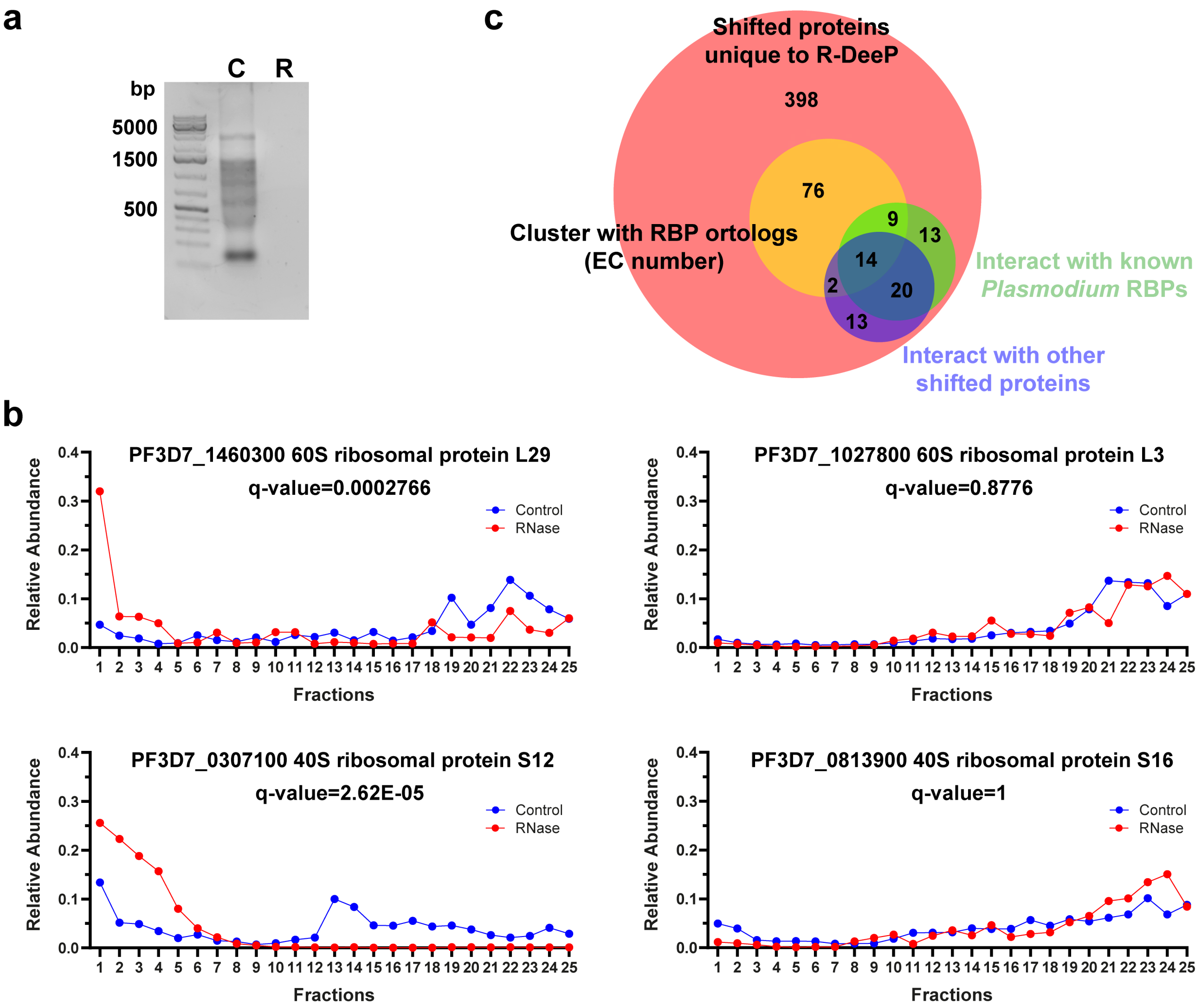

Supplementary Fig. 1. R-DeeP screening analysis. a. RNA quality check after enzymatic treatment in control and RNase conditions. The picture is representative of the two replicates. C = Control and R = RNase condition. b. Profiles of ribosomal proteins. Relative abundance of 60S and 40S ribosomal proteins illustrate RNase-shifted (left) and non-RNase-shifted (right) proteins. c. Venn diagram reporting the overlap between shifted proteins unique to the R-DeeP and proteins interacting with known RBPs or other shifted proteins (from protein-protein interaction study[23]), and with known RBP orthologs (from EC numbers).

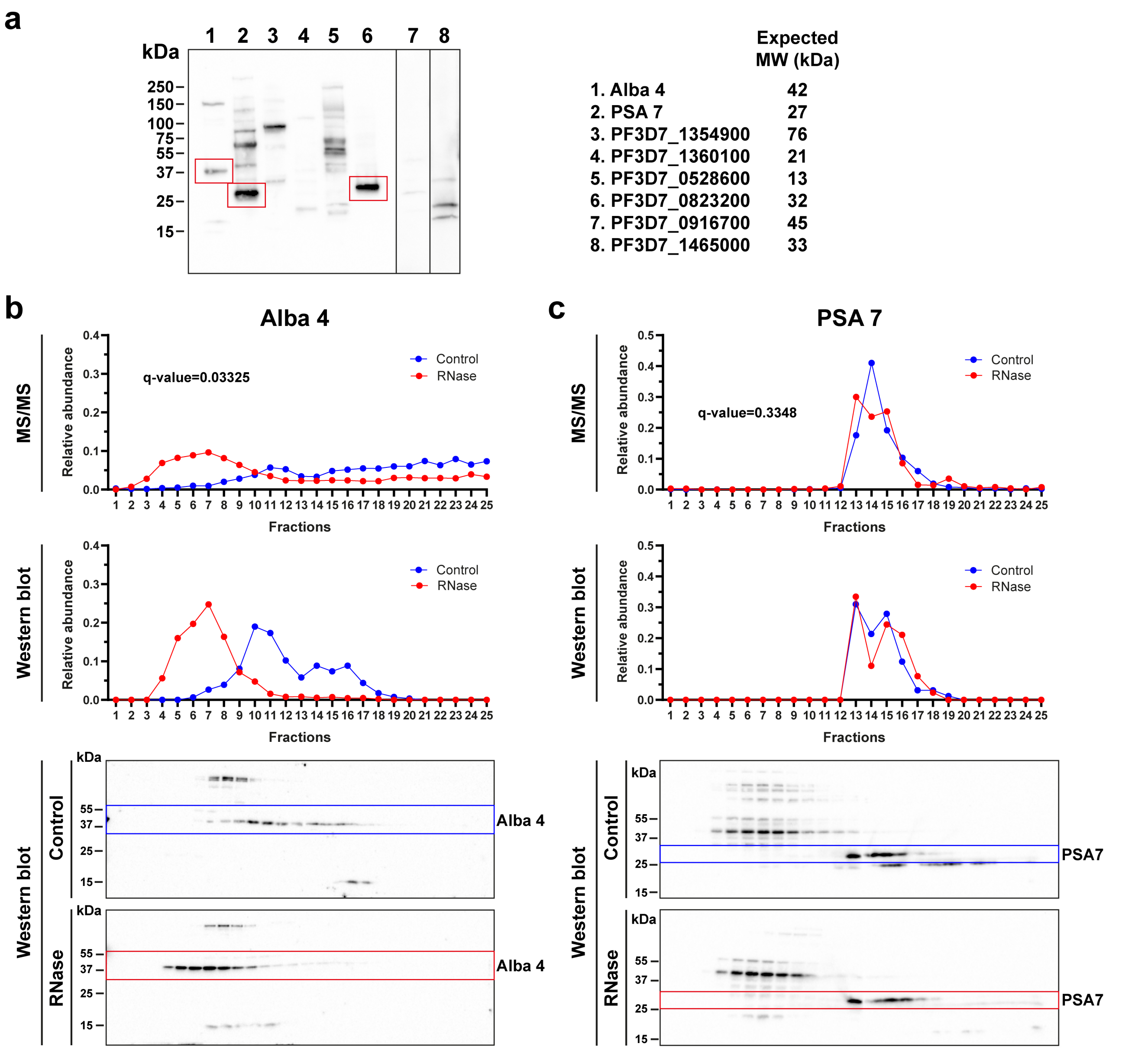

Supplementary Fig. 2. Validation of the R-DeeP screening. a. Western blot analysis of the eight custom antibodies after affinity-purification. The red squares highlight the expected proteins. b and c. Validation of the R-DeeP by western blot analysis. The mass spectrometry (MS/MS) data (top panel) were compared to the quantitative analyzes (center panel) of immunoblots obtained with anti-Alba 4 (b) and anti-PSA 7 (c) (bottom panel).

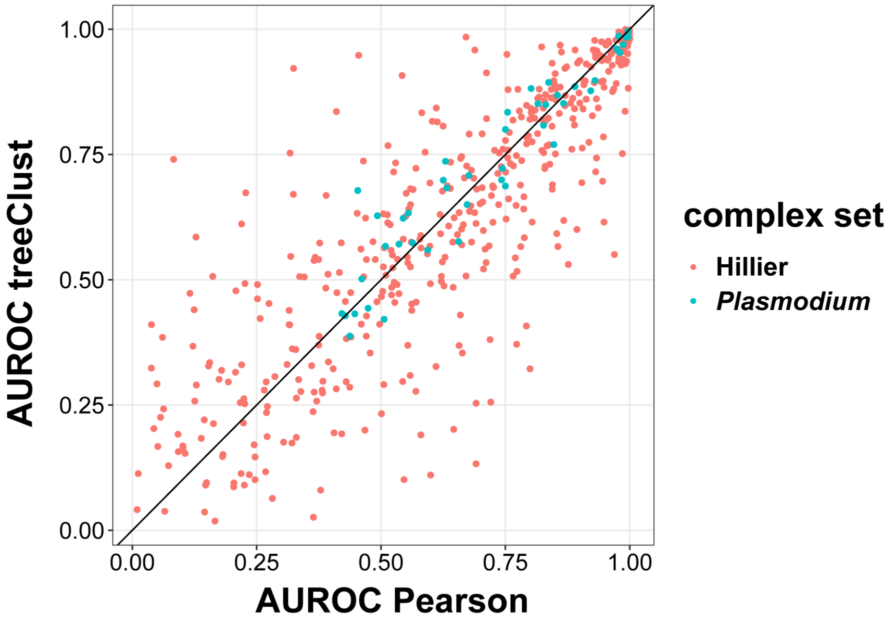

Supplementary Fig. 3. Comparison of protein similarity measures using Pearson correlation and treeClust dissimilarities. Each point corresponds to a protein complex, and the two axes represent AUROC values calculated using two different methods for ranking protein pairs.

Supplementary Fig. 4. Mean correlation of various *Plasmodium* complexes. For selected *Plasmodium* complexes, graphs show the mass spectrometry profiles of all partners in the control and RNase conditions (top part). The different control profiles were used to calculate the mean correlations which are represented in a heatmap (bottom part). The overall mean correlation and the RNase shift p-value for the respective complex are shown.

Pf60S

RNase shift p value: 0.0037

Missing proteins: PF3D7_1144300 PF3D7_0611700

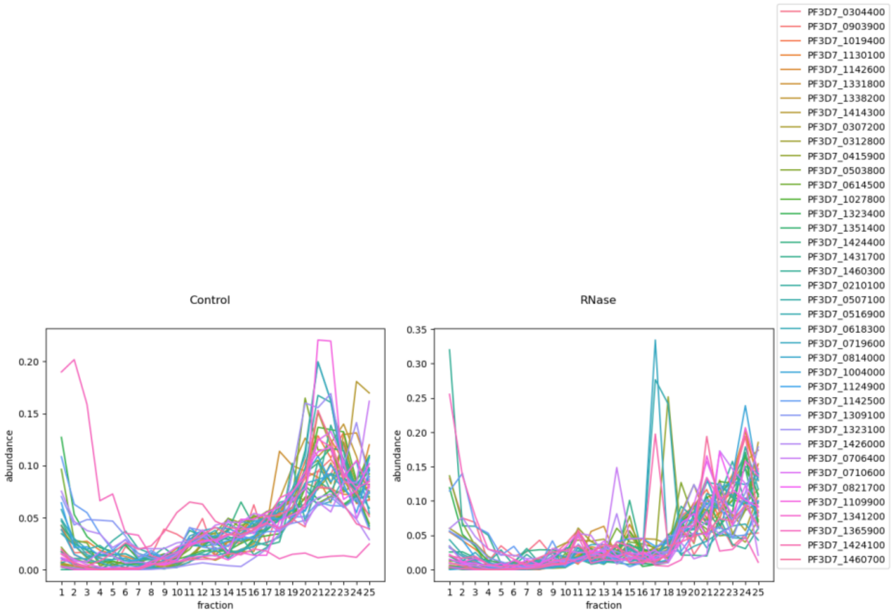

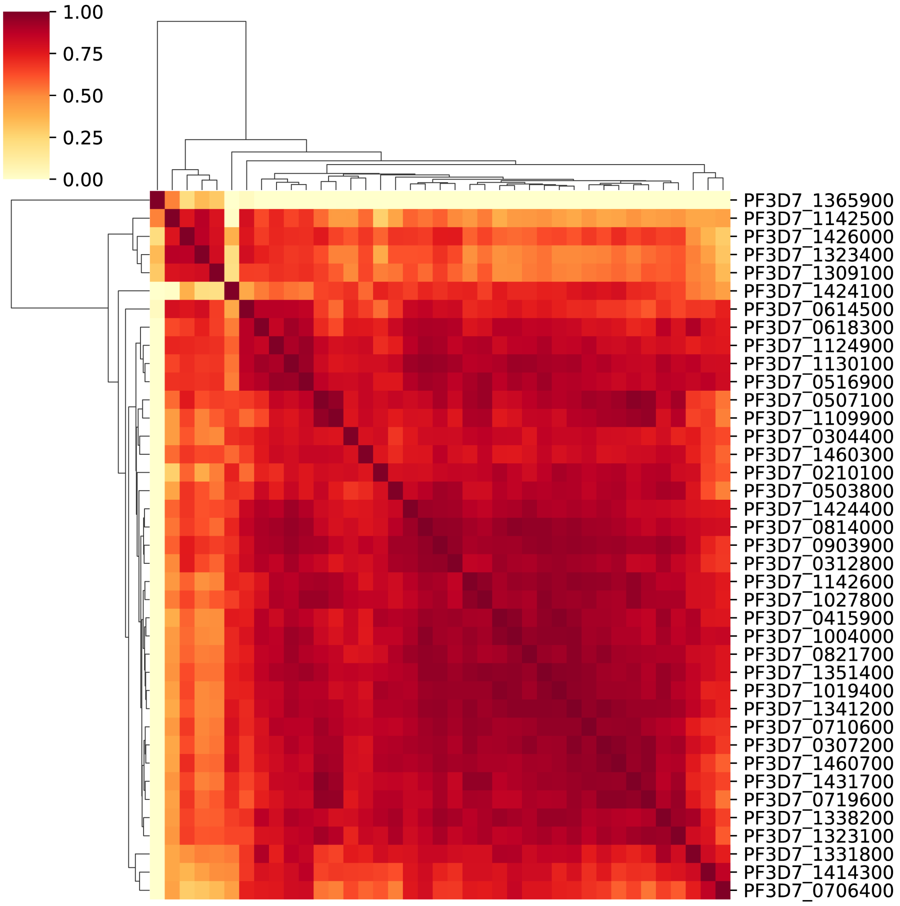

Mean correlation (control): 0.731

Pf40S

RNase shift p value: 5.8e-07

Missing proteins: PF3D7_0219200

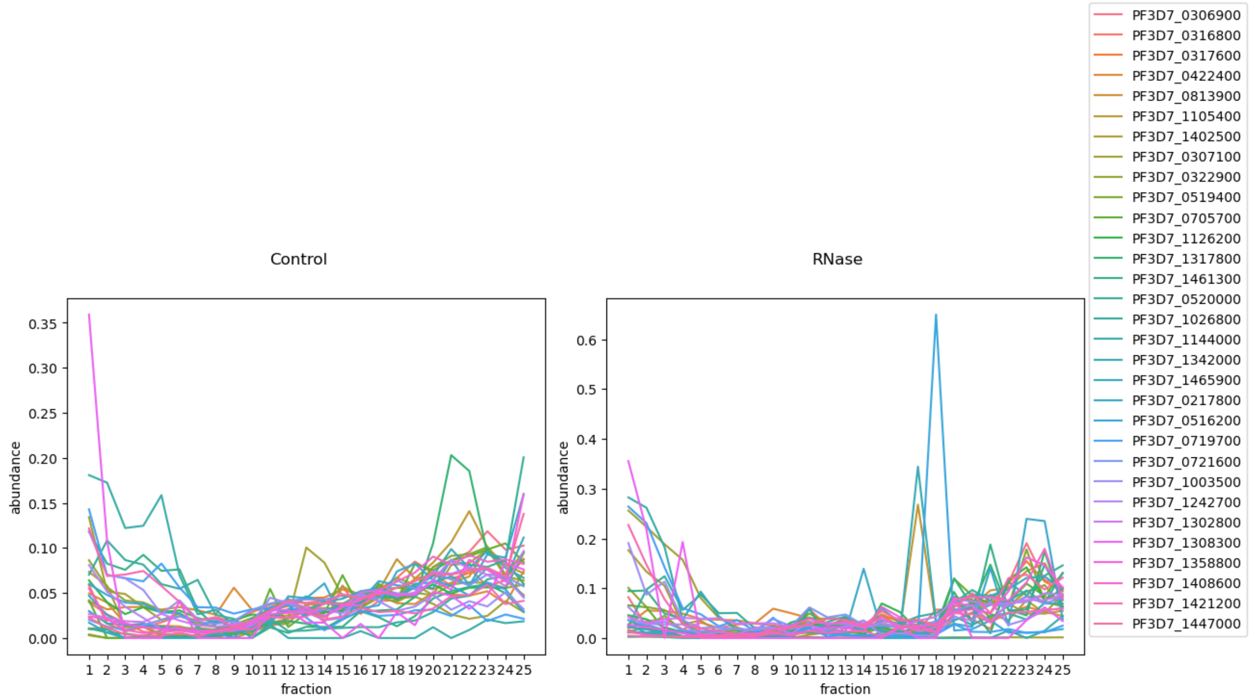

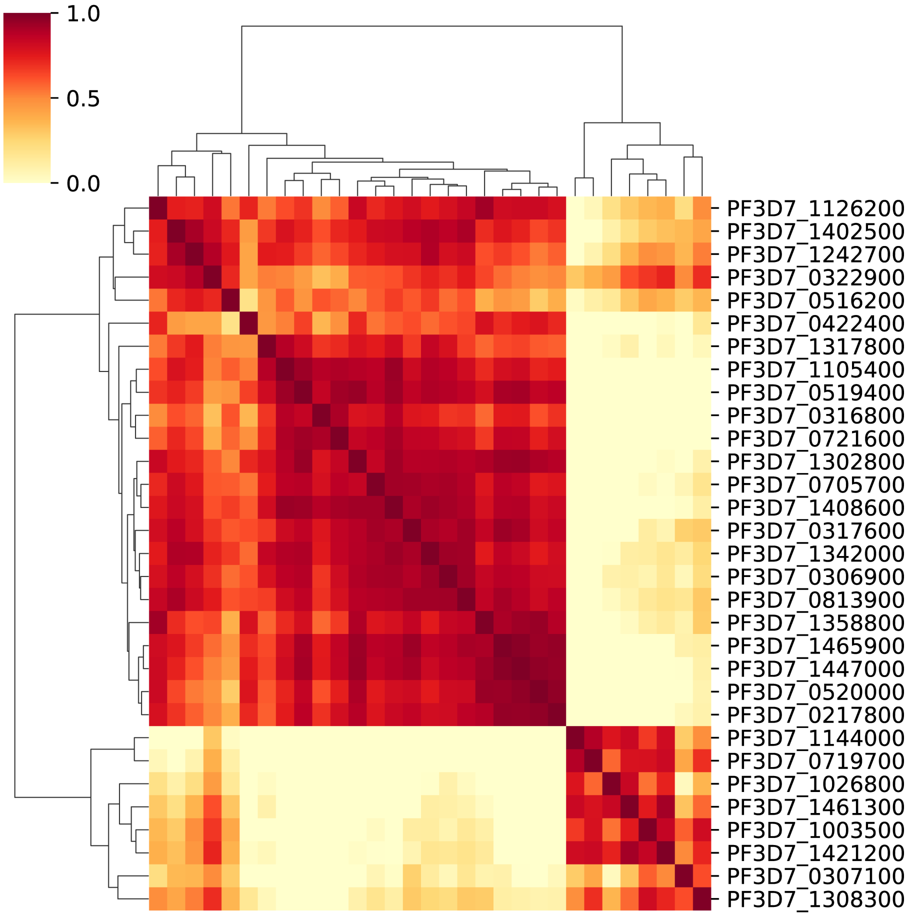

Mean correlation (control): 0.441

U2

RNase shift p value: 1.7e-11

Missing proteins:

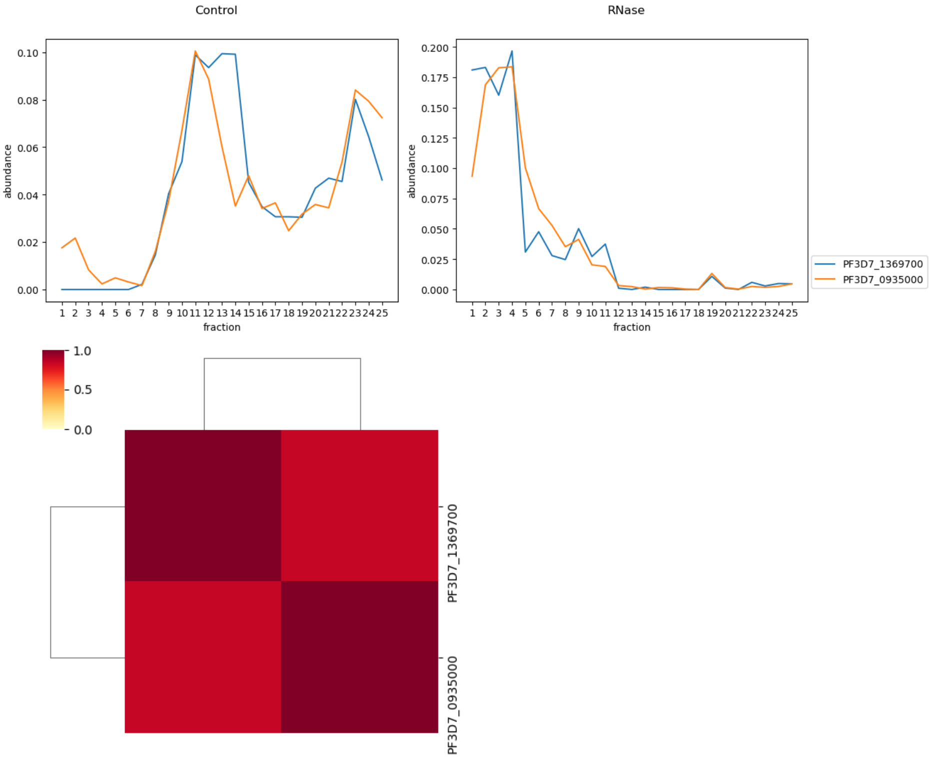

Mean correlation (control): 0.844

U6 (LSm)

RNase shift p value: 3.9e-11

Missing proteins:

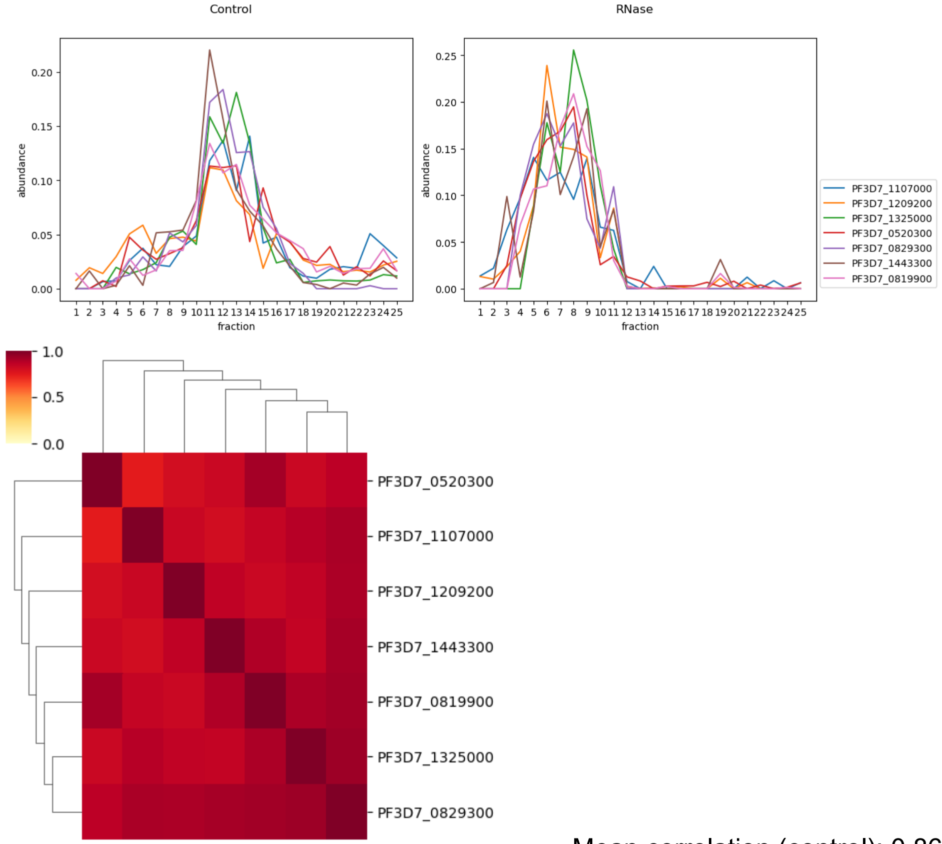

Mean correlation (control): 0.868

Prp19-CDC5

RNase shift p value: 0.0052

Missing proteins:

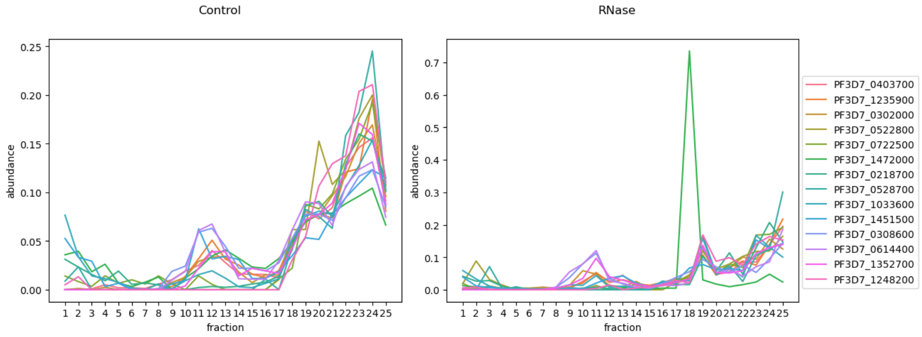

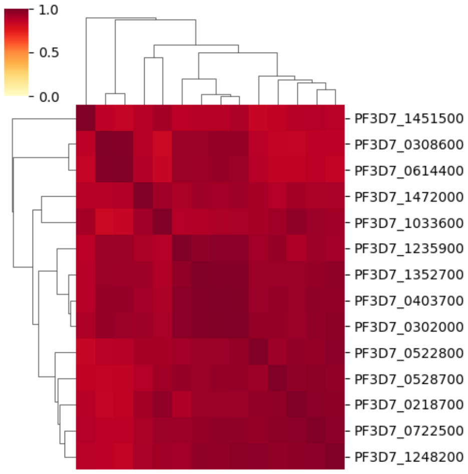

Mean correlation (control): 0.925

SM_proteins

RNase shift p value: 0.54

Missing proteins:

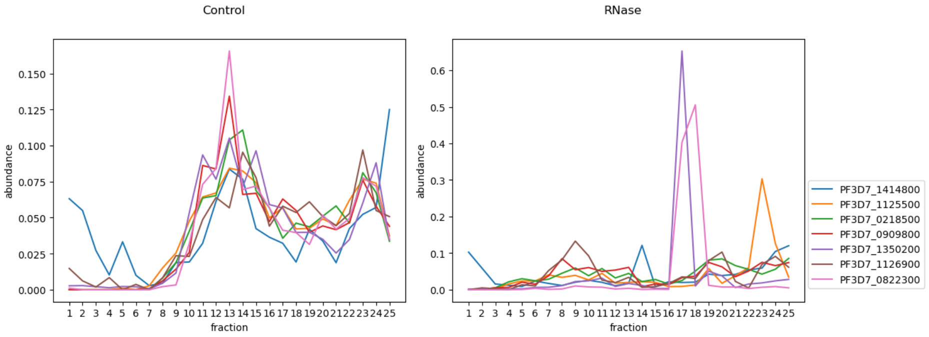

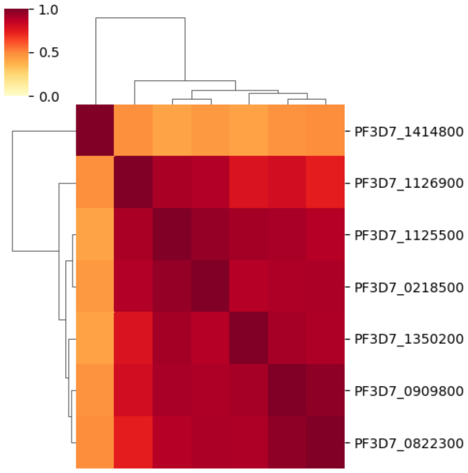

Mean correlation (control): 0.766

B_complex

RNase shift p value: 1.6e-45

Missing proteins:

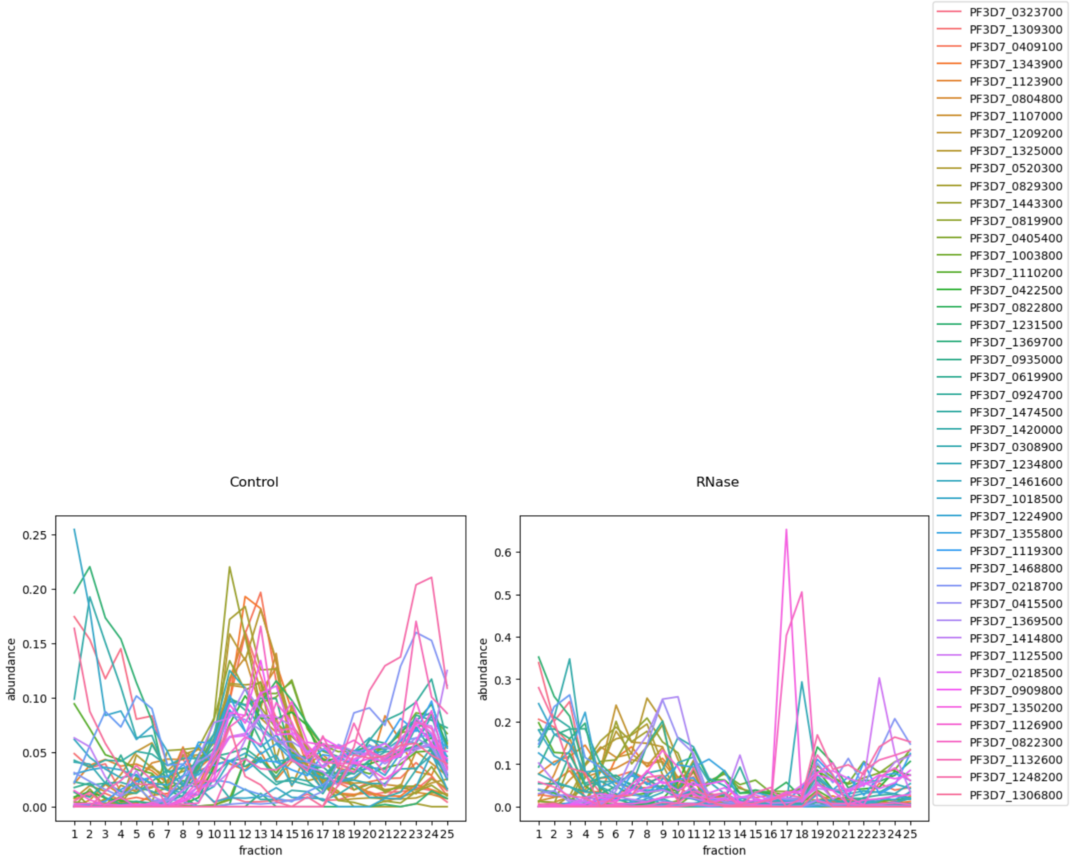

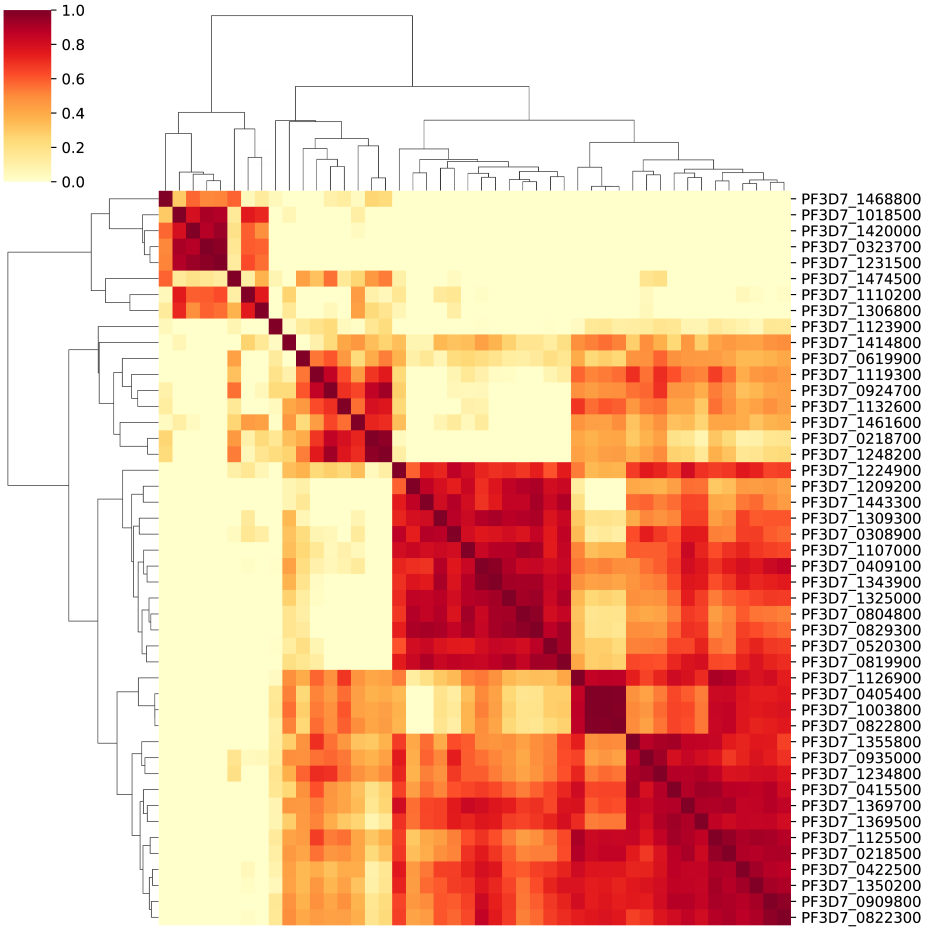

Mean correlation (control): 0.254

RNA-exosome

RNase shift p value: 0.042

Missing proteins:

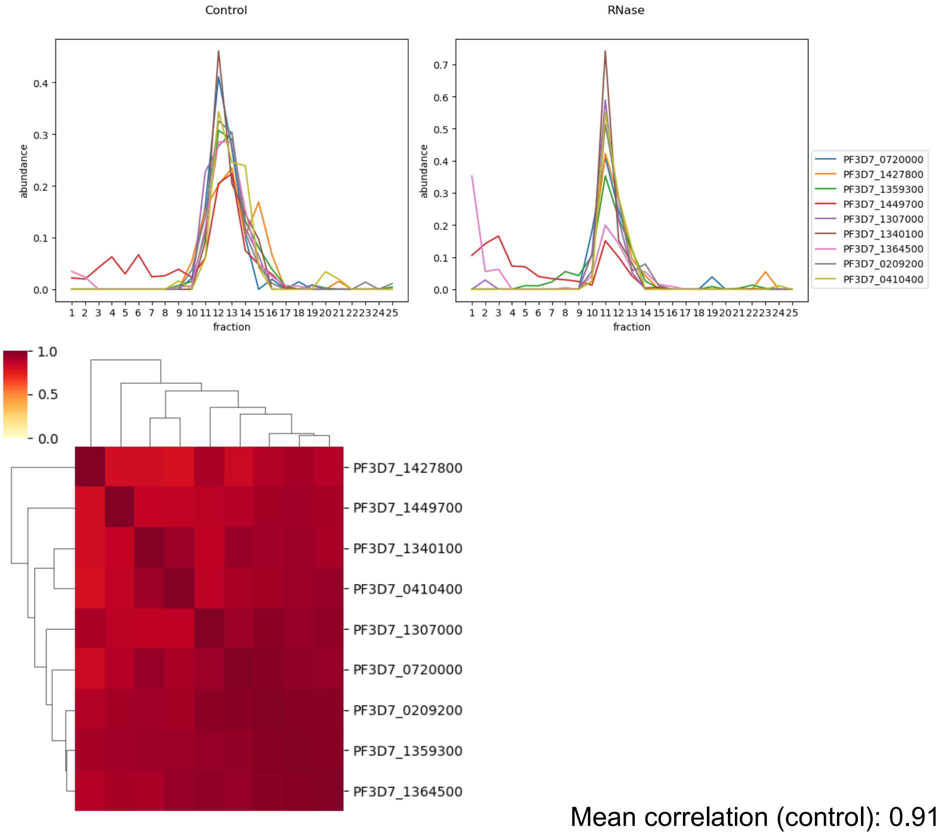

Mean correlation (control): 0.916

Mediator-complex

RNase shift p value: 0.012

Missing proteins: PF3D7_0505900 PF3D7_1475000 PF3D7_1363600 PF3D7_1463000

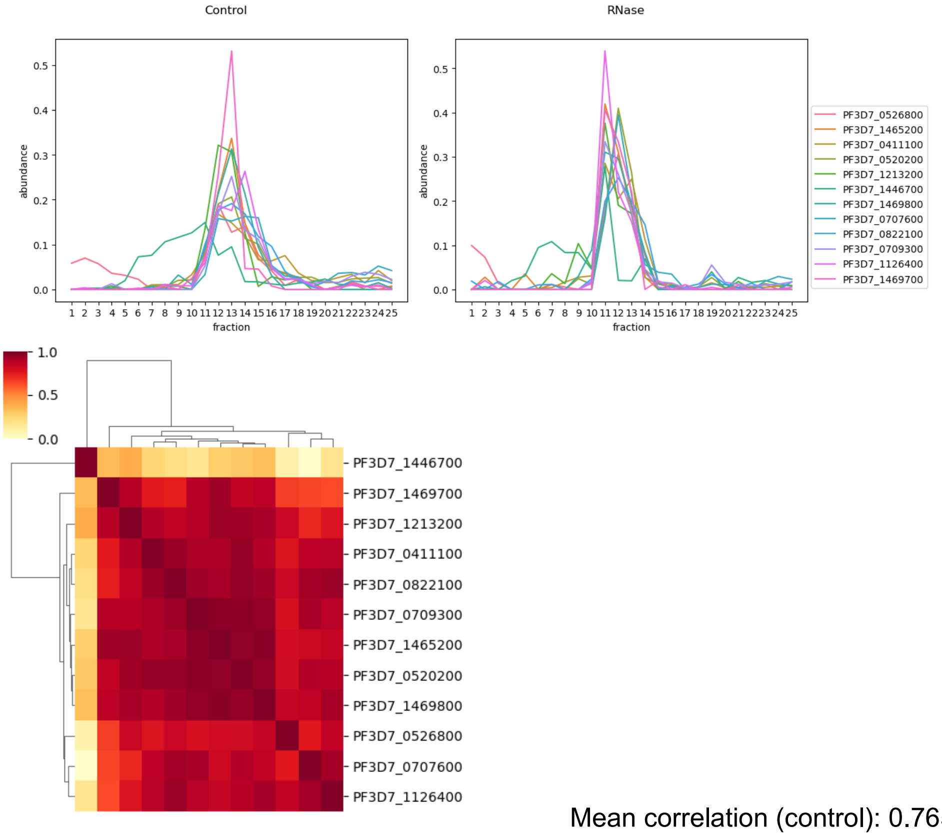

Mean correlation (control): 0.765

Mitochondrial-CII

RNase shift p value: 0.96

Missing proteins: PF3D7_0808450

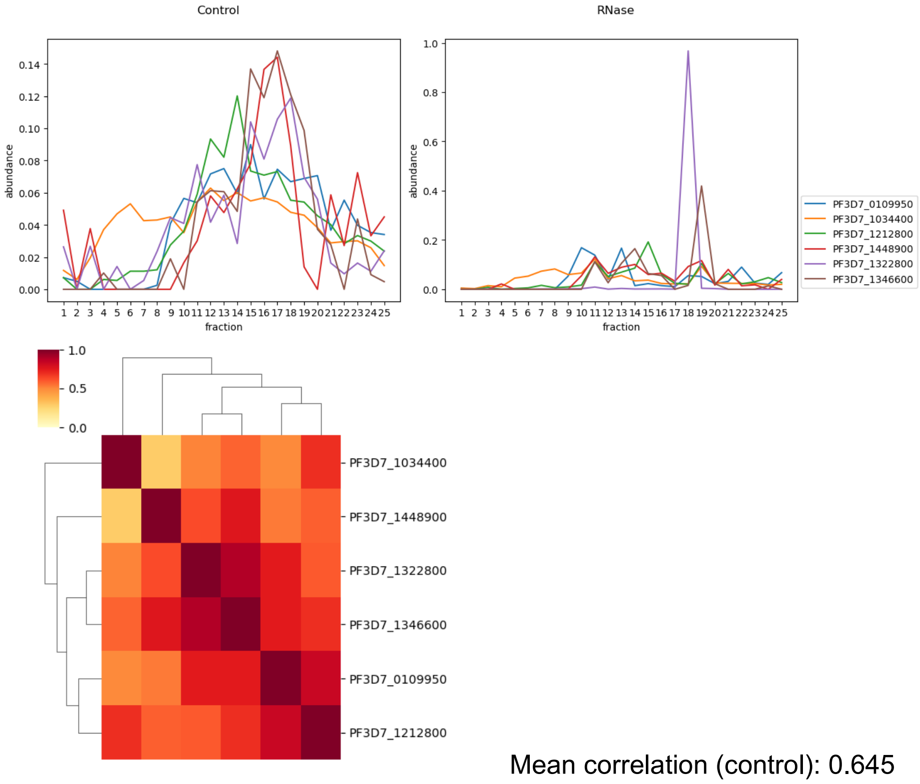

Mean correlation (control): 0.645

Mitochondrial-CIV

RNase shift p value: 0.75

Missing proteins: PF3D7_0809250 PF3D7_MIT01400 PF3D7_MIT02100

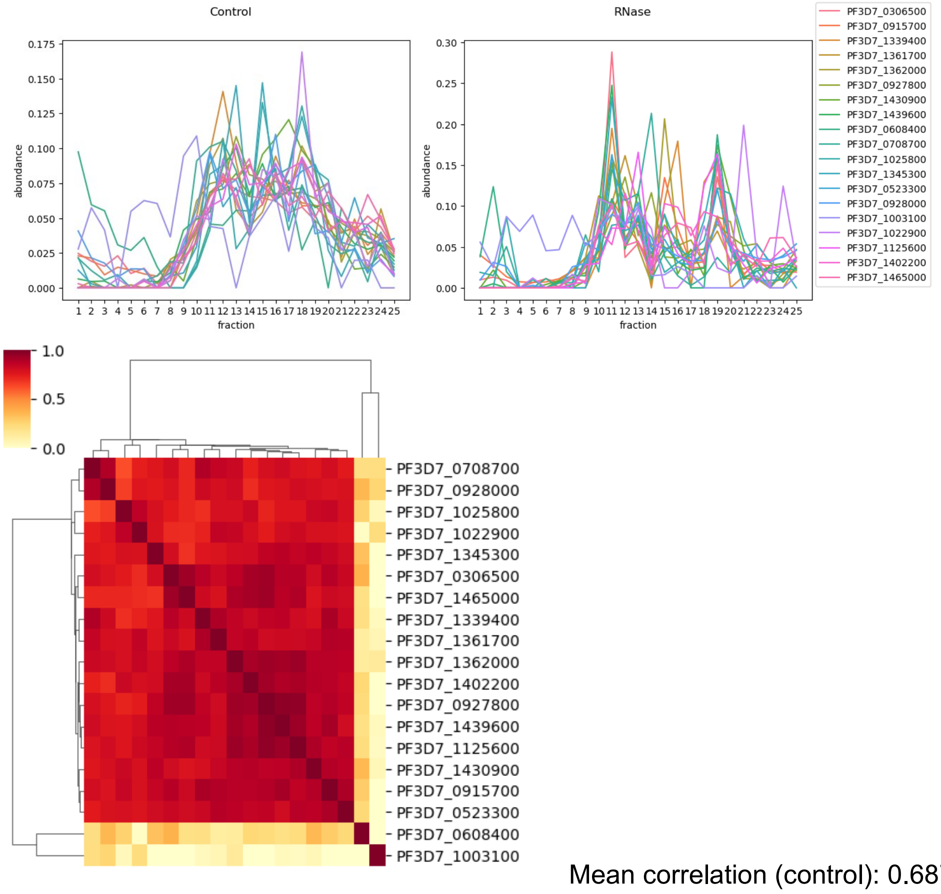

Mean correlation (control): 0.687

20S-Proteasome

RNase shift p value: 0.34

Missing proteins:

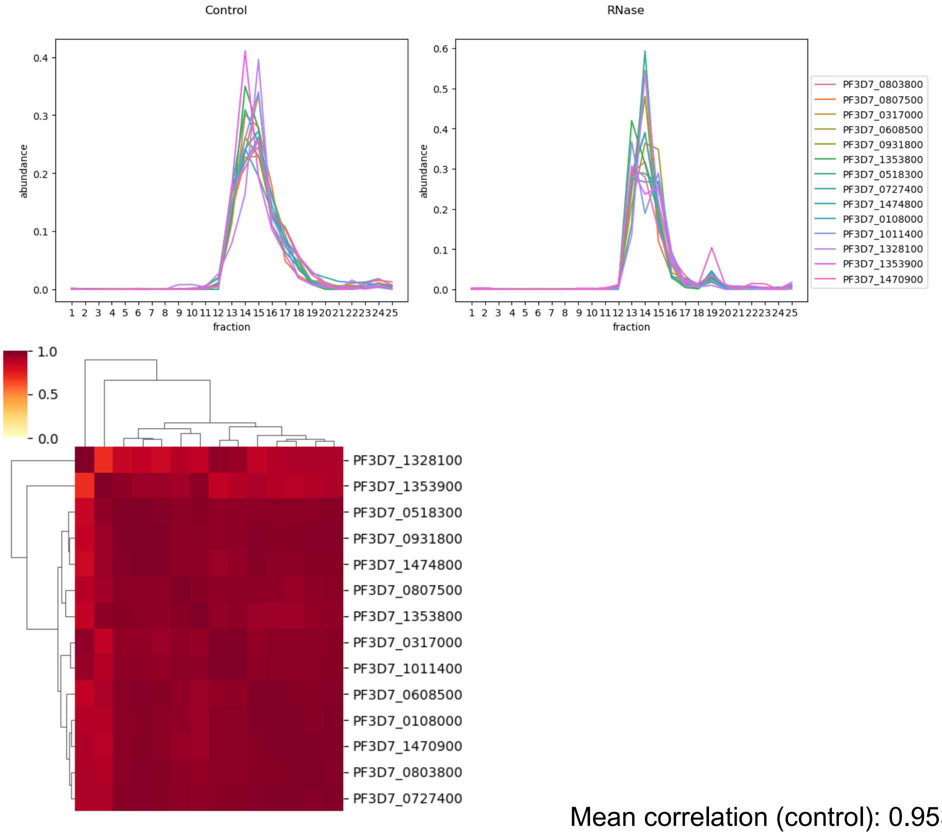

Mean correlation (control): 0.953

26S-Proteasome

RNase shift p value: 0.05

Missing proteins:

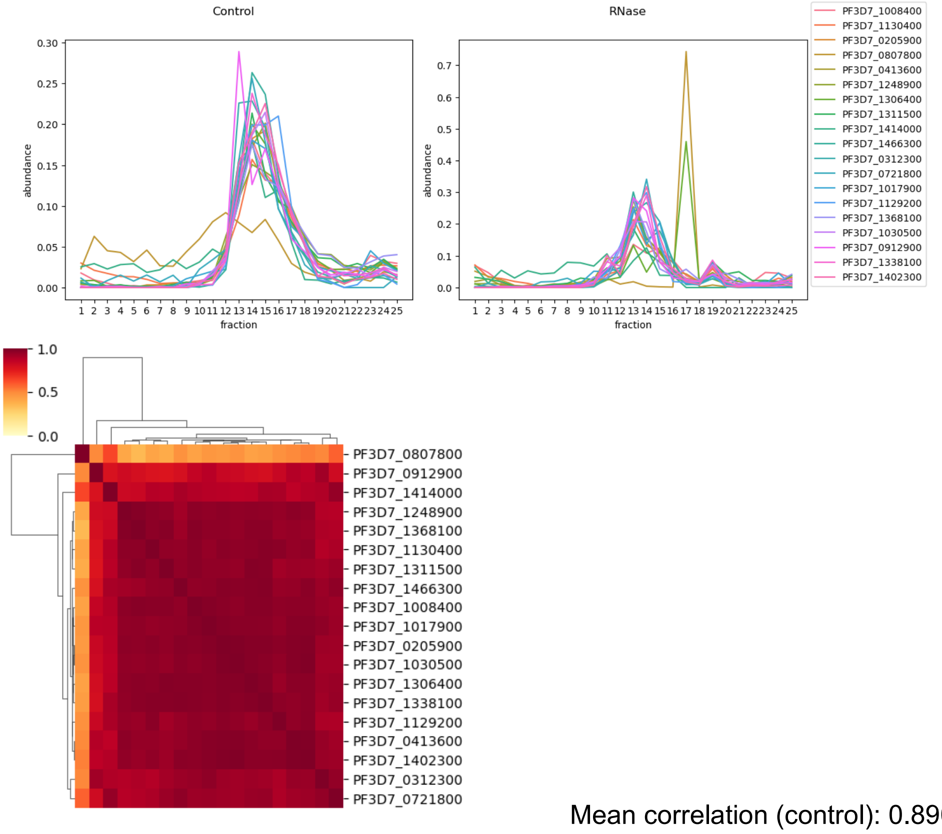

Mean correlation (control): 0.890

RNA-polymerase-I

RNase shift p value: 0.0067

Missing proteins:

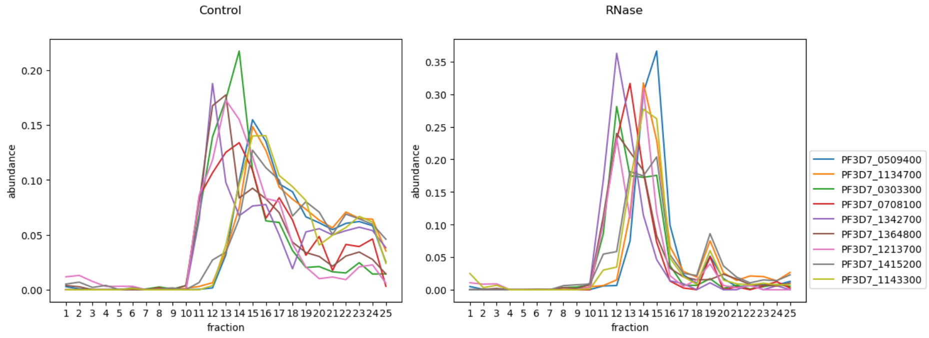

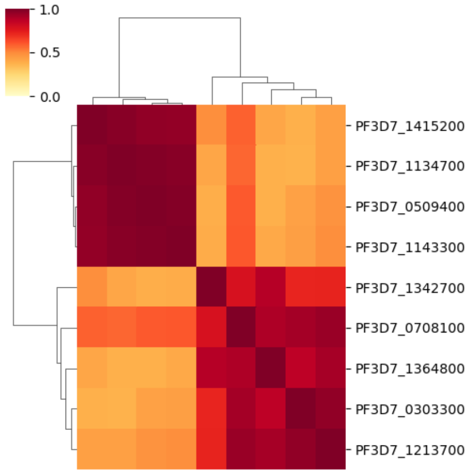

Mean correlation (control): 0.657

RNA-polymerase-III

RNase shift p value: 1.3e-12

Missing proteins:

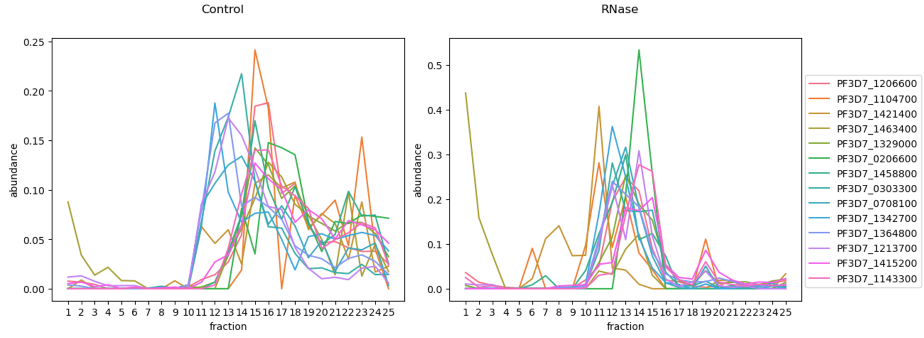

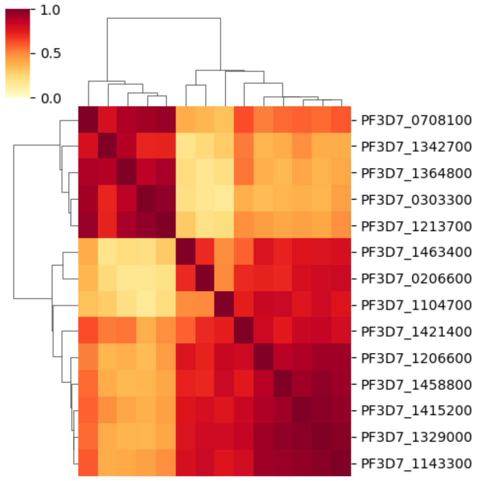

Mean correlation (control): 0.604

Supplementary Fig. 5. Mean correlation of *Plasmodium* protein clusters. For selected Hillier clusters[23], graphs show the mass spectrometry profiles of all partners in the control and RNase conditions (top part). Their respective cumulative distribution function profiles (CDF) are also displayed (second part). The different control profiles were used to calculate the mean correlations which are represented in a heatmap (bottom part). The overall mean correlation and the RNase shift p-value for the respective cluster are shown.

Cluster13

RNase shift p value: 0.003

Missing proteins: PF3D7_0611700

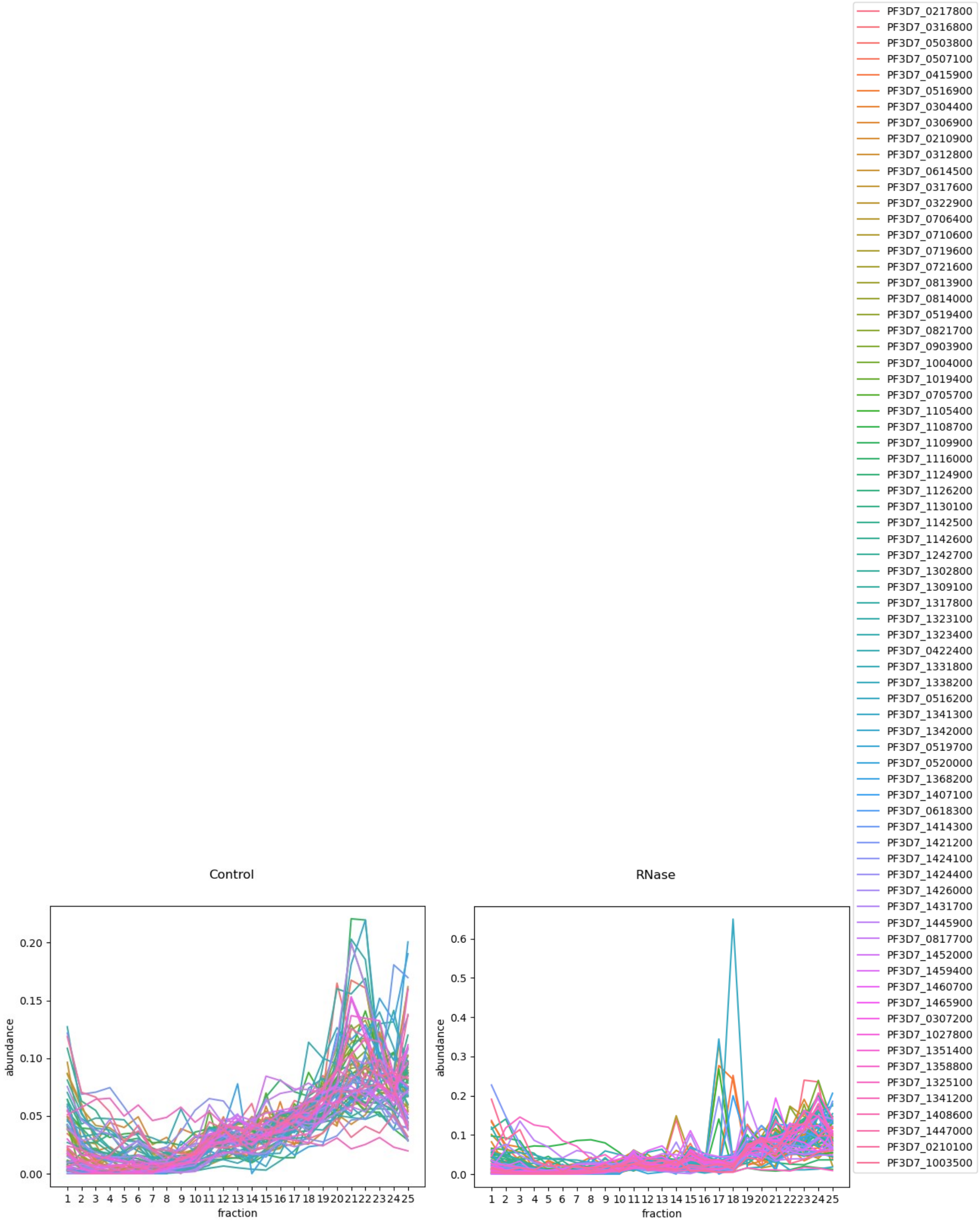

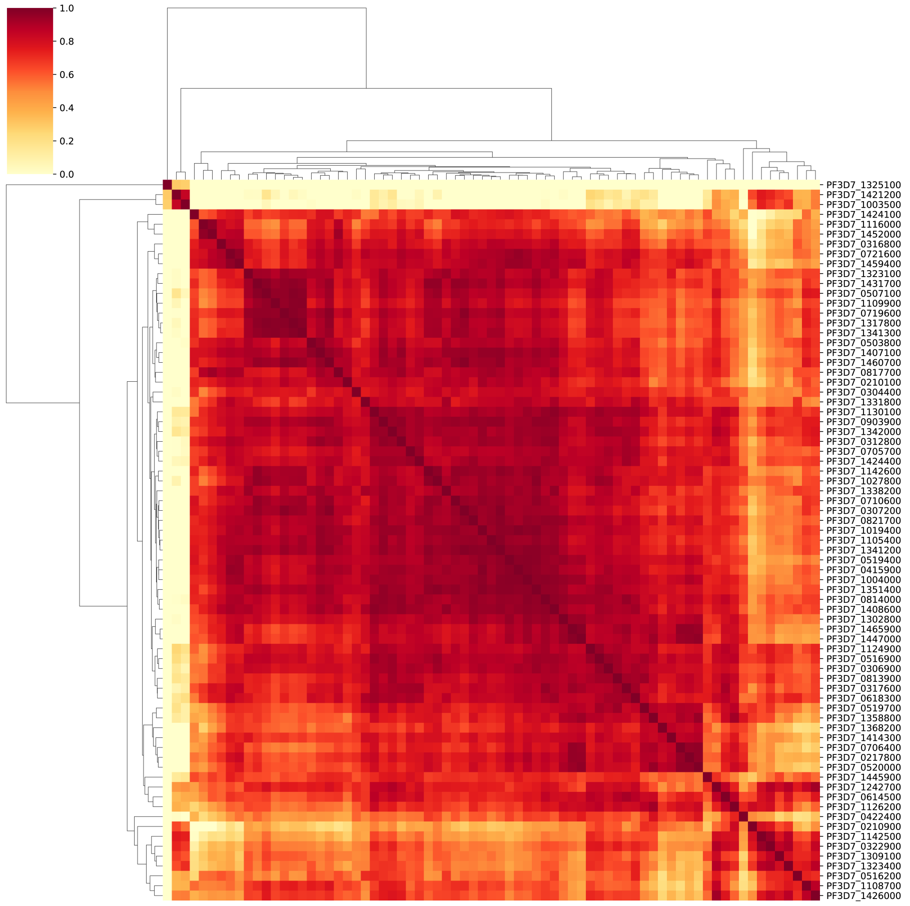

Mean correlation (control): 0.666

Cluster25

RNase shift p value: 0.093

Missing proteins:

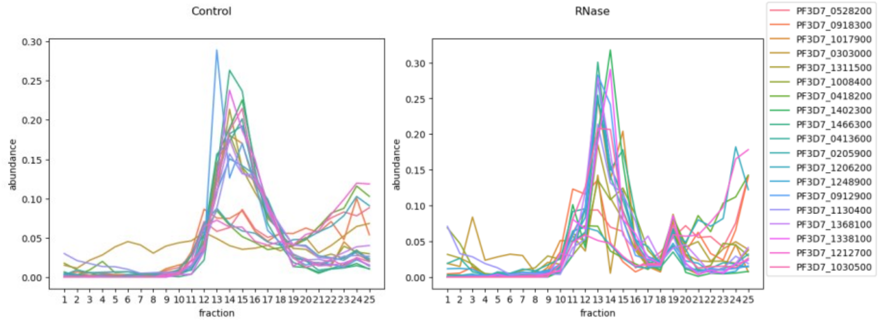

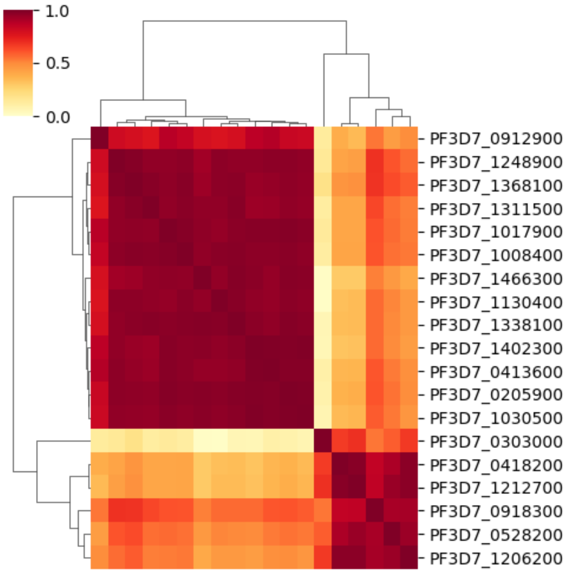

Mean correlation (control): 0.696

Cluster342

RNase shift p value: 0.041

Missing proteins:

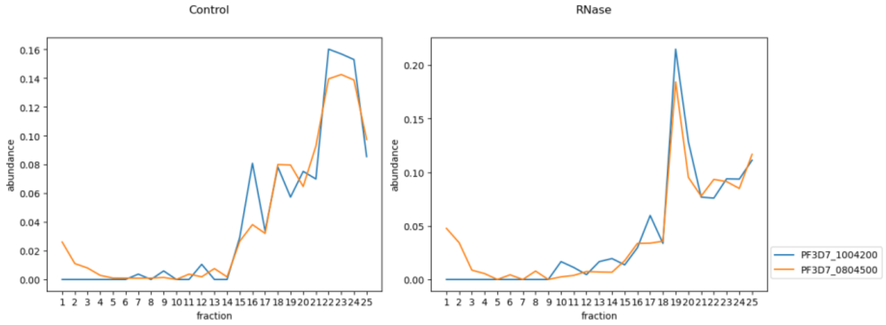

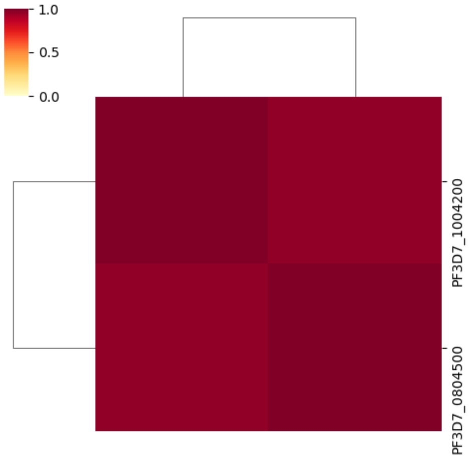

Mean correlation (control): 0.965

Cluster410

RNase shift p value: 0.00066

Missing proteins:

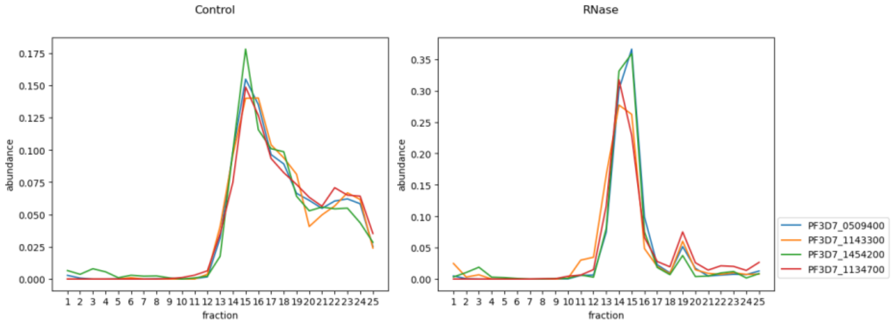

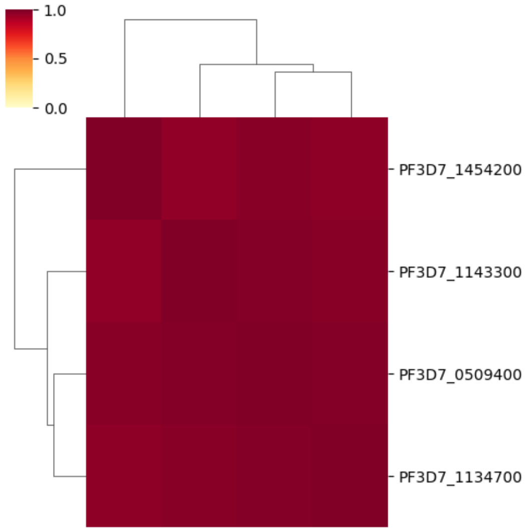

Mean correlation (control): 0.980

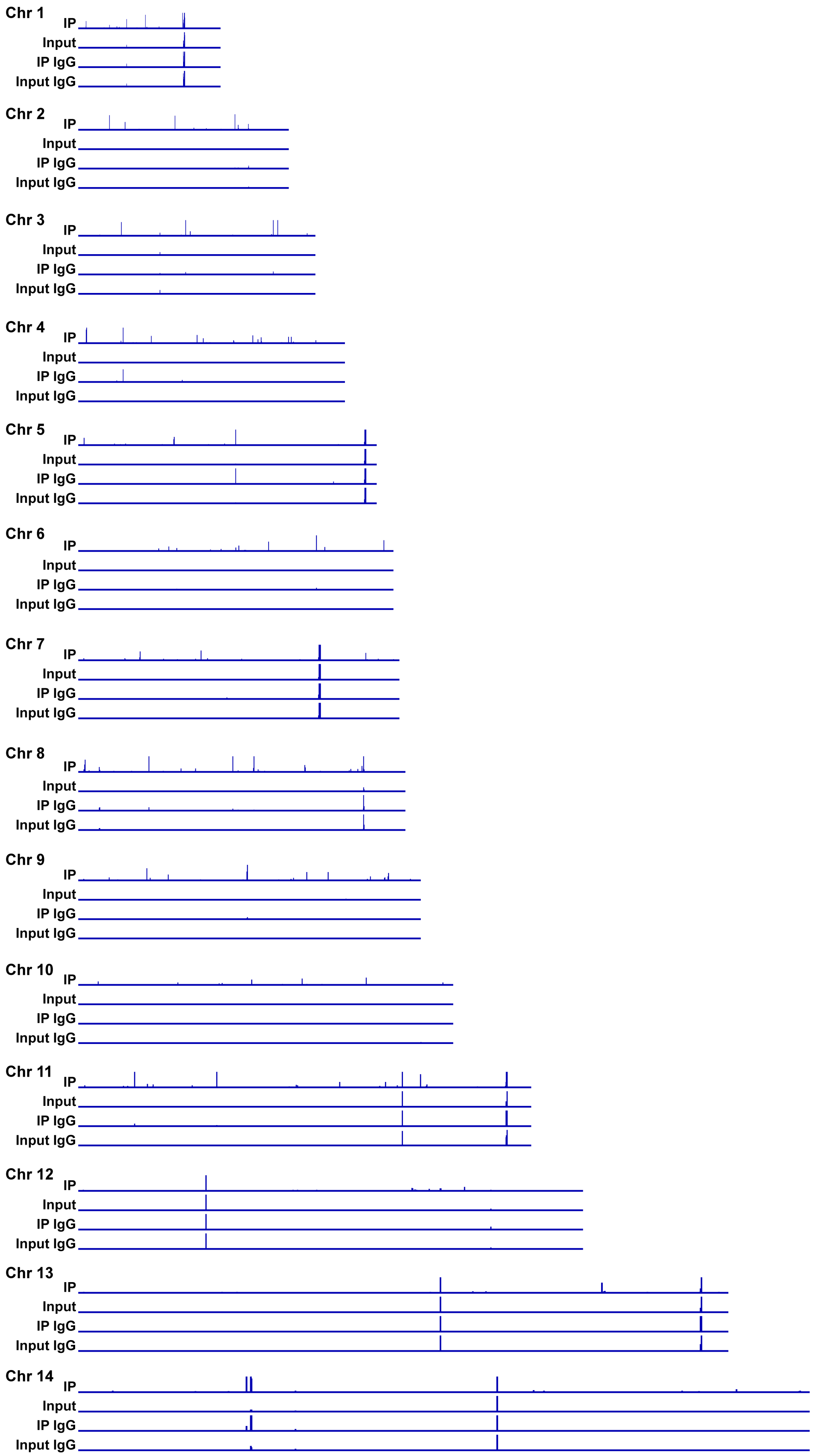

Supplementary Fig. 6. Summary of eCLIP-seq tracks. Representation of the eCLIP-seq peaks spanning the nuclear genome of *P. falciparum* for IP and Input samples of PF3D7_0823200 and IP and Input samples of IgG, in this respective order (scale 0-100). For each sample, both replicates were overlayed using Integrative Genomics Viewer (IGV).

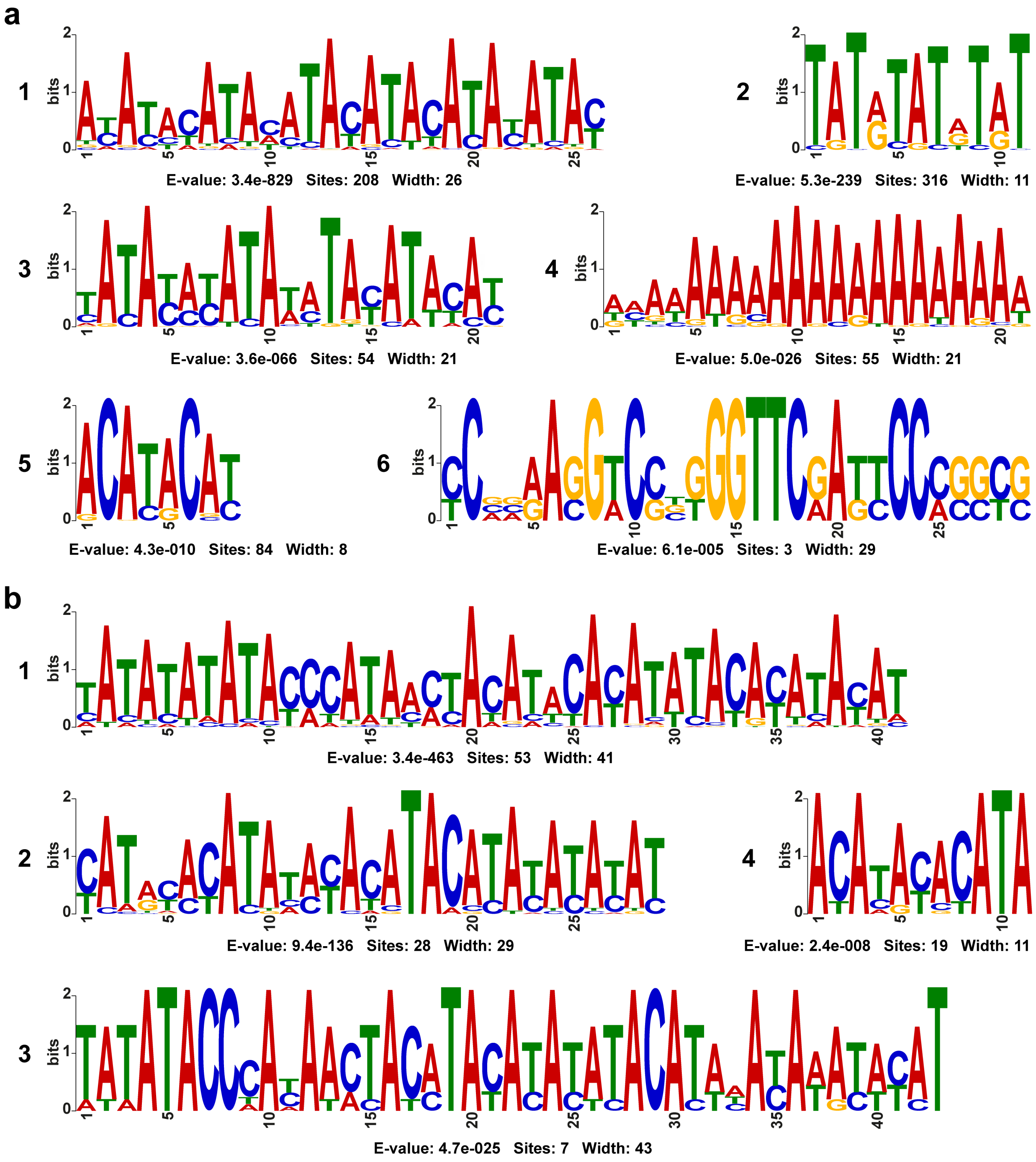

Supplementary Fig. 7. Sequence logos of the motifs identified by MEME Suite search. a. Significant binding motifs of PF3D7_0823200 identified using the 512 eCLIP-seq peaks. b. Significant binding motifs using the 56 peaks mapping on *var* genes. The number on the x-axis indicates the amino acid position in the motif. The height of letter represents the degree of conservation for each residue.
