# Supplementary material for: Proteome-Wide Identification of RNA-dependent proteins and an emerging role for RNAs in Plasmodium falciparum protein complexes
Source: Nat Commun. 2024 Feb 14;15:1365. doi: 10.1038/s41467-024-45519-1 (PMC10866993; doi:10.1038/s41467-024-45519-1)
Supplement: Supplementary file 3 — Inventory of Supporting Information [file 41467_2024_45519_MOESM3_ESM.docx]

Inventory of Supporting Information

**Manuscript #: Proteome-Wide Identification of RNA-Dependent Proteins and An Emerging Role for RNAs in *Plasmodium falciparum* Protein Complexes**

**Corresponding author name(s):** Karine Le Roch

| Please complete each of the Inventory Tables below to outline your Extended Data and Supplementary Information items.  There are four sections:   - *Extended Data* - *Supplementary Information: PDF Files* - *Supplementary Information: Additional Files* - *Source Data*   Each section includes specific instructions. Please complete these tables as fully as possible. We ask that you avoid using spaces in your file names, and instead use underscores, i.e.: Smith_ED_Fig1.jpg not Smith ED Fig1.jpg  Please note that titles and descriptive captions will only be lightly edited, so please ensure that you are satisfied with these prior to submission.  If you have any questions about any of the information contained in this inventory, please contact the journal. |
| --- |
|  |

1. **Extended Data**

**Complete the Inventory below for all Extended Data Figures and Tables**

- Keep Figure/Table titles to one sentence only
- Upload your files as ‘Figure Files’ in our Manuscript Tracking system
- File names should include the Figure/Table Number. i.e.: *Smith_ED_Fig1.jpg*
- Please be sure to include the file extension in the Filename. Note that Extended Data files (including tables) must be submitted as .jpg, .tif or .eps files *only*, and should be no more than 10MB
- All Extended Data item legends must be provided in the Inventory below and should not exceed 300 words each *(if possible)*
- Please include Extended Data *ONLY* in this table

| Figure or Table #  Please group Extended Data items by type, in sequential order. Total number of items (Figs. + Tables) must not exceed 10. | Figure/Table title  One sentence only | Filename  Whole original file name including extension. i.e.: Smith_ED_Fig1.jpg | Figure/Table Legend  If you are citing a reference for the first time in these legends, please include all new references in the main text Methods References section, and carry on the numbering from the main References section of the paper. If your paper does not have a Methods section, include all new references at the end of the main Reference list. |
| --- | --- | --- | --- |
| Choose an item. |  |  |  |
| Choose an item. |  |  |  |
| Choose an item. |  |  |  |
| Choose an item. |  |  |  |
| Choose an item. |  |  |  |
| Choose an item. |  |  |  |
| Choose an item. |  |  |  |
| Choose an item. |  |  |  |
| Choose an item. |  |  |  |
| Choose an item. |  |  |  |

***Do not insert additional rows - total number of Extended Data items must not exceed 10.***

1. **Supplementary Information:**
2. **PDF Files**

**Complete the Inventory below for all additional textual information and any additional Supplementary Figures, which should be supplied in one combined PDF file.**

- **Row 1:** A combined PDF containing any Supplementary Text, Discussion, Notes, Additional Supplementary Figures, Supplementary Protocols, simple tables, and all associated legends. **Only one such file is permitted**.
- **Row 2:** Nature Research’s Reporting Summary; if previously requested by the editor, please provide an updated Summary, fully completed, without any mark-ups or comments. **(Reporting Summaries are not required for all manuscripts.)**

**Note:** Please do not include a title page within your Supplementary Information file - a cover sheet that includes the title of your paper and a hyperlink to it will be automatically added while preparing your manuscript for publication

| Item | Present? | Filename  Whole original file name including extension. i.e.: Smith_SI.pdf. The extension must be .pdf | A brief, numerical description of file contents.  i.e.: *Supplementary Figures 1-4, Supplementary Discussion, and Supplementary Tables 1-4.* |
| --- | --- | --- | --- |
| Supplementary Information | Yes | Hollin_SI.pdf | Supplementary Figures 1-7 |
| Reporting Summary | Yes | Hollin-reporting-summary |  |
| Peer Review Information | Choose an item. | *OFFICE USE ONLY* |  |

1. **Additional Supplementary Files**

**Complete the Inventory below for all additional Supplementary Files that cannot be submitted as part of the Combined PDF.**

- Do not list Supplementary Figures in this table (see section 2A)
- Where possible, include the title and description within the file itself
- Spreadsheet-based tables & data should be combined into a workbook with multiple tabs, not submitted as individual files.
- Compressed files are acceptable where necessary. ZIP files are preferred.
- Please note that the *ONLY* allowable types of additional Supplementary Files are:

| - Supplementary Tables | - Supplementary Audio | - Supplementary Videos | - Supplementary Software |
| --- | --- | --- | --- |
| - Supplementary Code | - Supplementary Data, for example: - Source Data for Supplementary Figures - Raw NMR Data, Cryo-EM Data - Computational Data, Crystallographic Data, etc. | | |

| Type | Number  Each type of file (Table, Video, etc.) should be numbered from 1 onwards. Multiple files of the same type should be listed in sequence, i.e.: Supplementary Video 1, Supplementary Video 2, etc. | Filename  Whole original file name including extension. i.e.: *Smith_ Supplementary_Video_1.mov* | Legend or Descriptive Caption  Describe the contents of the file |
| --- | --- | --- | --- |
| Supplementary Data | Supplementary Data 1 | Supplementary_Data_1.xlsx | R-DeeP mass spectrometry dataset. |
| Supplementary Data | Supplementary Data 2 | Supplementary_Data_2.xlsx | Comparison of R-DeeP data and RNA-associated proteins. |
| Supplementary Data | Supplementary Data 3 | Supplementary_Data_3.xlsx | List of antibodies produced and used for western blot validation. |
| Supplementary Data | Supplementary Data 4 | Supplementary_Data_4.xlsx | Molecular weight analysis. |
| Supplementary Data | Supplementary Data 5 | Supplementary_Data_5.xlsx | Description and summary of protein complex analyzes. |
| Supplementary Data | Supplementary Data 6 | Supplementary_Data_6.xlsx | Comparative analysis of the protein PF3D7_0823200 in Apicomplexa. |
| Supplementary Data | Supplementary Data 7 | Supplementary_Data_7.xlsx | Proteins identified by MudPIT analysis after PF3D7_0823200 and IgG immunoprecipitations. |
| Supplementary Data | Supplementary Data 8 | Supplementary_Data_8.xlsx | Peak calling of eCLIP-seq samples using Piranha. |

***Add rows as needed to accommodate the number of files.***

**3. Source Data**

**Complete the Inventory below for all Source Data files.**

- Acceptable types of Source Data for Main Figures and Extended Data Figures or Tables are:
  - Statistical Source Data
    - Plain Text (ASCII, csv, TXT) or Excel formats only
    - Either one file for each relevant Figure, or a single file containing all source data, with clearly named tabs for each Figure/Extended Data Figure item
  - Full-length, unprocessed gels or blots
    - JPG, TIF, or PDF formats only
    - One file for each relevant Figure containing all supporting blots and/or gels, or a single file with clearly labeled gels or blots for each Figure/ED Figure item
- Source Data for Supplementary Figures is not allowed. Instead:
  - Include Unprocessed Gels or Blots for Supplementary Figures as additional Supplementary Figures to the main Supplementary Information PDF file.
  - Include Statistical Source Data for Supplementary Figures as ‘Supplementary Data’ files and list them in section 2B.
  - Please see [this example of Source Data](https://www.nature.com/articles/s41591-019-0505-4) in a publication.

| Parent Figure or Table | Filename  Whole original file name including extension. i.e.: *Smith_SourceData_Fig1.xls,* or *Smith_ Unmodified_Gels_Fig1.pdf* | Data description  i.e.: Unprocessed western Blots and/or gels, Statistical Source Data, etc. |
| --- | --- | --- |
| Source Data Fig. 1 |  |  |
| Source Data Fig. 2 |  |  |
| Source Data Fig. 3 |  |  |
| Source Data Fig. 4 |  |  |
| Source Data Fig. 5 |  |  |
| Source Data Fig. 6 |  |  |
| Source Data Fig. 7 |  |  |
| Source Data Fig. 8 |  |  |
| Source Data Extended Data Fig./Table 1 |  |  |
| Source Data Extended Data Fig./Table 2 |  |  |
| Source Data Extended Data Fig./Table 3 |  |  |
| Source Data Extended Data Fig./Table 4 |  |  |
| Source Data Extended Data Fig./Table 5 |  |  |
| Source Data Extended Data Fig./Table 6 |  |  |
| Source Data Extended Data Fig./Table 7 |  |  |
| Source Data Extended Data Fig./Table 8 |  |  |
| Source Data Extended Data Fig./Table 9 |  |  |
| Source Data Extended Data Fig./Table 10 |  |  |
