# Supplementary material for: Proteome-Wide Identification of RNA-dependent proteins and an emerging role for RNAs in Plasmodium falciparum protein complexes
Source: Nat Commun. 2024 Feb 14;15:1365. doi: 10.1038/s41467-024-45519-1 (PMC10866993; doi:10.1038/s41467-024-45519-1)
Supplement: Supplementary file 12 — Reporting Summary [file 41467_2024_45519_MOESM12_ESM.pdf]

nature portfolio

Corresponding author(s): Karine Le Roch

Last updated by author(s): Jan 2, 2024

Reporting Summary

Nature Portfolio wishes to improve the reproducibility of the work that we publish. This form provides structure for consistency and transparency in reporting. For further information on Nature Portfolio policies, see our Editorial Policies and the Editorial Policy Checklist.

Statistics

For all statistical analyses, confirm that the following items are present in the figure legend, table legend, main text, or Methods section.

n/a	Confirmed	
☐	☒	The exact sample size (*n*) for each experimental group/condition, given as a discrete number and unit of measurement
☐	☒	A statement on whether measurements were taken from distinct samples or whether the same sample was measured repeatedly
☐	☒	The statistical test(s) used AND whether they are one- or two-sided<br>*Only common tests should be described solely by name; describe more complex techniques in the Methods section.*
☒	☐	A description of all covariates tested
☐	☒	A description of any assumptions or corrections, such as tests of normality and adjustment for multiple comparisons
☐	☒	A full description of the statistical parameters including central tendency (e.g. means) or other basic estimates (e.g. regression coefficient) AND variation (e.g. standard deviation) or associated estimates of uncertainty (e.g. confidence intervals)
☐	☒	For null hypothesis testing, the test statistic (e.g. *F*, *t*, *r*) with confidence intervals, effect sizes, degrees of freedom and *P* value noted<br>*Give P values as exact values whenever suitable.*
☒	☐	For Bayesian analysis, information on the choice of priors and Markov chain Monte Carlo settings
☒	☐	For hierarchical and complex designs, identification of the appropriate level for tests and full reporting of outcomes
☐	☒	Estimates of effect sizes (e.g. Cohen's *d*, Pearson's *r*), indicating how they were calculated

Our web collection on statistics for biologists contains articles on many of the points above.

Software and code

Policy information about availability of computer code

Data collection	Image Lab software (version 5)<br>Keyence BZ-X800
Data analysis	GraphPad Prism (version 9)<br>ImageJ (version 1.53i)<br>DeepVenn (https://www.deepvenn.com/)<br>UpSet plot (https://gehlenborglab.shinyapps.io/upsetr/)<br><br>High-throughput sequencing:<br>IGV (version 2.7.2)<br>Piranha (version 1.2.1)<br>R package ChIPseeker (version 1.24.0)<br><br>Proteomics:<br>Proteowizard<br>Crux (version 3.2-46bb0c1)<br>Param-Medic (version 0.1)<br>Percolator (version 3.6)<br>RAWDistiller (version 1.0)<br>ProLuCID (version 1.3.3)<br>DTASelect (version 1.9)

Contrast (version 1.9)
NSAF7 (version 0.0.1) https://github.com/tzw-wen/kite/tree/master/windowsapp/NSAF7x64

Protein complex analysis:
sklearn.decomposition package (version 1.3.2)

For manuscripts utilizing custom algorithms or software that are central to the research but not yet described in published literature, software must be made available to editors and reviewers. We strongly encourage code deposition in a community repository (e.g. GitHub). See the Nature Portfolio guidelines for submitting code & software for further information.

Data

Policy information about availability of data

All manuscripts must include a data availability statement. This statement should provide the following information, where applicable:

- Accession codes, unique identifiers, or web links for publicly available datasets
- A description of any restrictions on data availability
- For clinical datasets or third party data, please ensure that the statement adheres to our policy

Gene Ontology datasets used in this study can be accessed from PlasmoDB website (https://plasmodb.org/plasmo/app). eCLIP-seq datasets generated in this study have been deposited in the NCBI Sequence Read Archive under accession number PRJNA949221 (https://www.ncbi.nlm.nih.gov/bioproject/PRJNA949221). The R-DeeP and IP-MS datasets have been deposited in the MassIVE repository with identification number MSV000091565 (https://massive.ucsd.edu/ProteoSAFe/dataset.jsp?task=c1704f223dda4177a932d7de7e7ea63e) and MSV000091228 (https://massive.ucsd.edu/ProteoSAFe/dataset.jsp?task=47d09718782e490ebaf90b0d66a744ea), respectively. Original data underlying this manuscript generated at the Stowers Institute can be accessed from the Stowers Original Data Repository (http://www.stowers.org/research/publications/LIBPB-2374).

Research involving human participants, their data, or biological material

Policy information about studies with human participants or human data. See also policy information about sex, gender (identity/presentation), and sexual orientation and race, ethnicity and racism.

Reporting on sex and gender	This research did not involve human participants.
Reporting on race, ethnicity, or other socially relevant groupings	Not applicable.
Population characteristics	Not applicable.
Recruitment	Not applicable.
Ethics oversight	Not applicable.

Note that full information on the approval of the study protocol must also be provided in the manuscript.

Field-specific reporting

Please select the one below that is the best fit for your research. If you are not sure, read the appropriate sections before making your selection.

☒ Life sciences ☐ Behavioural & social sciences ☐ Ecological, evolutionary & environmental sciences

For a reference copy of the document with all sections, see nature.com/documents/nr-reporting-summary-flat.pdf

Life sciences study design

All studies must disclose on these points even when the disclosure is negative.

Sample size	For the R-DeeP experiments the number of parasites was calculated to obtain at least 2 mg of soluble proteins per condition. This amount of proteins is described as optimal for R-DeeP experiments (PMID: 32094787).<br>The number of parasites for IP-MS and eCLIP-seq was chosen according to standards in the field and as previously validated (PMID: 35277503).<br><br>Sample size and statistical analysis are indicated in the Methods section.
Data exclusions	No data were excluded.
Replication	The R-DeeP experiments were performed in duplicate. IFAs and western blot analyses were done in at least two independent experiments. IP-MS was performed in triplicate. eCLIP-seq experiments were performed in duplicate. All attempts at replication were successful.
Randomization	Protein extracts for R-DeeP experiments were randomly asssigned to control and RNase conditions. The eight custom antibodies were validated on random protein extracts. For the validation of the R-DeeP using western blot analysis, samples were distributed according to

their respective experimental groups (control vs RNase). For IP-MS and eCLIP-seq, parasites were randomly assigned to anti-PF3D7_0823200 or IgG antibodies.

Blinding	Mass spectrometry samples (R-DeeP and IP-MS) and sequencing of the eCLIP-seq libraries were blinded for our collaborators. Blinding was not relevant for IFAs since there was only one experimental condition. The western blot experiments were not blinded since the relative amount of each protein was detected and normalized by Image lab (BioRad).

Reporting for specific materials, systems and methods

We require information from authors about some types of materials, experimental systems and methods used in many studies. Here, indicate whether each material, system or method listed is relevant to your study. If you are not sure if a list item applies to your research, read the appropriate section before selecting a response.

Materials & experimental systems

n/a	Involved in the study	
☐	☒	Antibodies
☐	☒	Eukaryotic cell lines
☒	☐	Palaeontology and archaeology
☒	☐	Animals and other organisms
☒	☐	Clinical data
☒	☐	Dual use research of concern
☒	☐	Plants

Methods

n/a	Involved in the study	
☒	☐	ChIP-seq
☒	☐	Flow cytometry
☒	☐	MRI-based neuroimaging

Antibodies

Antibodies used

Peptide antigens were designed to target the C-terminal region of:
PF3D7_0528600, PF3D7_1354900, PF3D7_1360100, PF3D7_0823200, PF3D7_0916700, PF3D7_1347500, PF3D7_1353900 and PF3D7_1465000.
They were used to immunize two rabbits and antisera from day 72 post-immunization were collected (Thermo Fisher Scientific).

Immunoblots:
Primary antibodies: anti-PF3D7_0823200, anti-PF3D7_1347500 and anti-PF3D7_1353900 were used at 1:50. Anti-Plasmodium aldolase antibody (ab207494) was used at 1:10,000.
Secondary antibody: HRP-labeled Goat anti-Rabbit IgG (H + L) at 1:10,000 (NovexTM, A16104).

Immunofluorescence Assays:
Anti-PF3D7_0823200 was used at 1:100 followed by Donkey anti-Rabbit Alexa Fluor 568 at 1:2,000 (Invitrogen, A10042).

IP-MS and eCLIP-seq:
Anti-PF3D7_0823200 at 1:100 and purified Rabbit IgG at 1:100 (MP Biomedicals, 0855944).

Validation

Custom antibody specificity was tested by western blot analysis on total P. falciparum protein extract.
Anti-Plasmodium aldolase antibody (ab207494) was validated by Abcam for immunoblot of P. falciparum samples. This antibody was previously validated for western blot using P. falciparum samples (PMID: 35277503).

Eukaryotic cell lines

Policy information about cell lines and Sex and Gender in Research

Cell line source(s)	Plasmodium falciparum NF54 and 3D7 strains were provided by the Malaria Research and Reference Reagent Resource Center (MR4).
Authentication	Authenticated by the provider.
Mycoplasma contamination	Not tested.
Commonly misidentified lines (See ICLAC register)	No commonly misidentified lines were used in this study.

Plants

Seed stocks	This research did not involve plants.
Novel plant genotypes	This research did not involve plants.
Authentication	This research did not involve plants.
